# Outcome of illustrated information leaflet on correct usage of asthma-metered dose inhaler

**DOI:** 10.4102/phcfm.v11i1.2079

**Published:** 2019-08-21

**Authors:** Wendy Wrench, Lynette van Dyk, Sunitha Srinivas, Ros Dowse

**Affiliations:** 1 Faculty of Pharmacy, Rhodes University, Grahamstown, South Africa

**Keywords:** primary health care, asthma, metered dose inhaler use, patient education, illustrated leaflet, pictograms, limited literacy patients

## Abstract

**Background:**

Research globally has shown that metered dose inhaler (MDI) technique is poor, with patient education and regular demonstration critical in maintaining correct use of inhalers. Patient information containing pictorial aids improves understanding of medicine usage; however, manufacturer leaflets illustrating MDI use may not be easily understood by low-literacy asthma patients.

**Aim:**

To develop and evaluate the outcome of a tailored, simplified leaflet on correct MDI technique in asthma patients with limited literacy skills.

**Setting:**

A rural primary health care clinic in the Eastern Cape, South Africa.

**Methods:**

Pictograms illustrating MDI steps were designed to ensure cultural relevance. The design process of the leaflet was iterative and consultative involving a range of health care professionals as well as patients. Fifty-five rural asthma patients were recruited for the pre-post design educational intervention study. Metered dose inhaler technique was assessed using a checklist, and patients were then educated using the study leaflet. The principal researcher then demonstrated correct MDI technique. This process was repeated at follow-up 4 weeks later.

**Results:**

The number of correct steps increased significantly post intervention from 4.6 ± 2.2 at baseline to 7.9 ± 2.7 at follow-up (*p* < 0.05). Statistically significant improvement of correct technique was established for 10 of the 12 steps. Patients liked the pictograms and preferred the study leaflet over the manufacturer leaflet.

**Conclusion:**

The tailored, simple, illustrated study leaflet accompanied by a demonstration of MDI technique significantly increased correct MDI technique in low-literacy patients. Patients approved of the illustrated, simple text leaflet, and noted its usefulness in helping them improve their MDI technique.

## Introduction

Patients who are diagnosed with a non-communicable disease are expected to adopt and learn new and sometimes complex behaviours. This includes accepting the need for change, and learning and implementing new skills after a brief interaction with a health care professional (HCP).^1^ For patients with pulmonary disease, this often occurs when experiencing an asthma exacerbation, at which time they may not be able to assimilate information given because of being distressed and unable to breathe easily. However, many HCPs assume that patients will implement their instructions in order to improve their health and if this does not occur, the patient may be blamed and be regarded as incompetent or recalcitrant.^2^ Patient-centric discussions to promote self-management of asthma facilitate the development of a partnership between patient and HCP, which enables the acquisition of knowledge, confidence and skills, and in which patients are encouraged to participate in decisions about their treatment and given the opportunity to express their expectations and concerns.^3,4^

The administration of corticosteroids and bronchodilators via inhalation is considered the optimal route for appropriate drug delivery for treatment of bronchial asthma, potentially significantly reducing asthma hospitalisations and improving symptom control.^5^ Learning and maintaining the correct inhaler technique is a complex process and is related to asthma control, type of inhaler device and patient motivation.^4,6^ However, incorrect use of oral inhaler devices, particularly the metered dose inhaler (MDI), is one of the most common causes that hinders better asthma control.^4,5,7,8,9,10^ This is reportedly a universal problem as noted in research literature emanating from diverse sources such as Italy,^11^ Iran,^12^ United States (US),^13^ Saudi Arabia,^14,15^ Trinidad,^16^ Spain,^17^ France,^18^ Nigeria^19^ and the Netherlands,^20^ representing a significant societal and health-economic burden.^4^

Despite more than 40 years of awareness of the high frequency of this problem, it still has not been resolved.^8,9^ This suggests that more time should be invested by HCPs in educating and training patients on how to use their inhalers correctly^4,5,8,9,10^ and, in turn, supporting HCP practice by identifying ways in which effective inhaler technique education can be delivered.^4,6,10^ Successful asthma management is reportedly 10% medication and 90% education.^21^ If patients are to successfully self-manage, they must be educated about their asthma,^3^ as patient education has been shown to be effective in managing bronchospasm, thereby improving asthma control and health-related quality of life (HRQOL).^22^ Low confidence in inhaler usage has been associated with lower adherence and poor health status.^23^

When educating patients, it is essential to consider the potential barriers of literacy, culture and language. In developing countries, provider–patient communication is often challenged by linguistic, socio-economic, educational and cultural differences.^24,25^ If the patient’s primary language is different to that of the HCP and/or if the patient has low-literacy skills, the patient may be hesitant or embarrassed to admit to not understanding instructions.^26^ A South African study with isiXhosa-speaking adults and children with asthma found that culture and language barriers negatively affected health care.^27^ Patients and carers were unable to understand questions posed by HCPs and felt that they could not ask questions which resulted in unreliable patient histories, difficulty in explaining what asthma is, and counselling on correct use of medicines. The use of medical jargon by HCPs and its inclusion in written patient information further compounds communication problems.^27^

Raynor et al.,^28^ in their comprehensive review on the role and effectiveness of written medicines information to patients, concluded that current information does not fulfil patient needs. They suggested that patients should be involved in the design process, and that findings of information design and content should be used to improve quality. A written information leaflet must convey essential and relevant information in a clear, understandable and easily readable form to the target patient group, with attention paid to content, design and layout to optimise its usability.^25,29,30,31^ Any visual material such as pictograms must be relevant to the text, meaningful, include images familiar to the viewer and must be logically located. A well-designed leaflet with good readability and visual material can promote self-management of chronic conditions.^29,30,31^

The aim of this research was to develop a simple, user-friendly information leaflet describing correct MDI usage, which contained simple text and pictograms, to evaluate the outcome of the leaflet combined with a technique demonstration on correct MDI use, and to assess its acceptability in rural patients with asthma who have limited literacy skills.

## Methods

### Study design and setting

A pre-post intervention study design was adopted in which each patient acts as his or her own control. Patients were recruited from a rural primary health care (PHC) clinic in the Eastern Cape province of South Africa. Health care services are generally provided by a nurse-managed PHC clinic, and patients with chronic conditions such as asthma are required to attend the clinic every 4 weeks for a medical check-up and to collect a 28-day supply of medicines. Medical history and prescribed medicines are recorded in a ‘health passport’, a booklet in which each visit to a health care facility is recorded. This is retained by the patient who is required to present it at each visit.

### Study population and sampling strategy

The majority of the population in the Eastern Cape are black Africans (87.5%), with 72.2% living in rural areas. For inclusion in this study, patients had to be 18 years or older, dependent on public sector health care facilities, diagnosed with asthma, prescribed an MDI (salbutamol and/or beclomethasone) for at least 1 month, and be either English or isiXhosa-speaking. The exclusion criterion for patients in this study was involvement in any other asthma educational intervention during the study duration.

Sample size was determined using the *Z* test for proportions power calculation. With a significance level of 5% and the power of the test = 0.80, the calculated sample size for a one-tailed test was 54. The sample size of 55 used in the analysis resulted in the power of the test to be 0.81. Sample size was limited by the number of patients with asthma attending this isolated rural PHC clinic.

### Design of the information leaflet

A third of the population in the Eastern Cape has a maximum of primary schooling and may have difficulty reading and comprehending written information.^32^ Guidelines for designing written health information and health-related visual materials for low-literacy readers were consulted during the design and development of the study leaflet. They advised consideration of language and culture, simplification of text, avoidance of medical terms, use of short sentences, personalisation of information and inclusion of simple, familiar visuals (or pictograms).^25,29,30,31,33^ Previous research by W.W. and S.S. also informed leaflet design, as well as personal experience of W.W. of living with asthma and expertise developed while working in public sector hospitals and PHC clinics.

The research team (W.W., S.S., and R.D.) worked with a graphic artist who designed the initial version of the 10 pictograms included in the patient information leaflet (PIL). The initial design was a cost-effective, black and white, double-sided Z-fold design using landscape A4 paper. In acknowledgement of the prevailing limited literacy skills in the target population, text was simplified, the number of words limited and any medical jargon was avoided. In an iterative process thereafter, HCPs (doctors, nurses and pharmacists), academic pharmacist researchers and patients from the target population were consulted to offer feedback on the visual characteristics of the pictograms and to assess appropriateness and understandability of the text. These included informal interviews with nurses, as well as general discussion and feedback during pharmacy and therapeutics committee (PTC) meetings at one hospital and at one PHC clinic which informed further modifications to produce the second version of the leaflet.

A further round of discussions with seven patients, nine community health workers, five pharmacists and one PTC meeting generated further comment after which the pilot leaflet was produced. This version was quantitatively evaluated in a pilot study involving nine asthma patients from the target population, with results and feedback from this study informing minor modifications to the leaflet and the questionnaire, resulting in the final version of both. A comprehensive report of the design process is available.^34^

**FIGURE 1:**
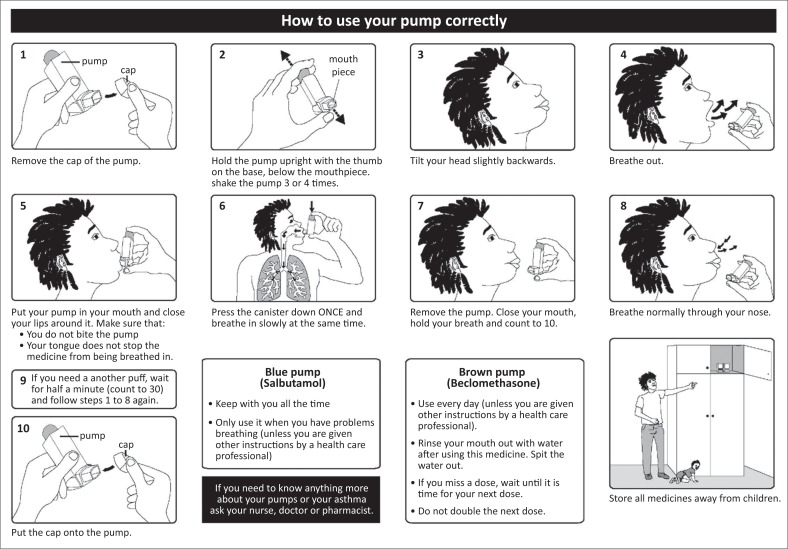
English version of the illustrated leaflet describing use of the metered dose inhaler.

The leaflet was translated into isiXhosa by an African languages expert at Rhodes University and back-translated by a different person to check for accuracy of translation. See Figure 1 for the English version of the leaflet.

### Intervention

In this pre-post study design, all recruited patients were recipients of the intervention. The intervention consisted of a structured assessment of inhaler technique followed by a demonstration of the correct technique by the primary researcher (W.W.) and took between 10 minutes and 20 minutes. The demonstration and patient education session were supported and facilitated by reference to the study leaflet on MDI use.

### Data collection

The following data were collected during individual interviews using a questionnaire: demographics, medical history relating to asthma and prescribed medicines, assessment of MDI technique, ability to read the industry-generated MDI instruction leaflet and acceptability of the study leaflet.

To address the language barrier, an interpreter from the target population was recruited who had prior experience in communicating with patients and who was trained for this study. All individual interviews were conducted by the primary researcher (W.W.) with the assistance of the interpreter. Following a consultation with a nurse, patients received their medications and were referred to W.W. Potential participants were informed about the study via the interpreter who also assisted patients with signing the consent form. Patients were encouraged to ask questions at any stage of the interview.

After the collection of personal data, patients were asked to demonstrate their MDI technique with two inhalations from a placebo MDI. No prior instructions or prompts were given. Inhaler technique was evaluated according to a 12-step checklist:

- Remove mouth piece cap.
- Hold pump upright with thumb on base, below mouthpiece.
- Shake pump.
- Tilt head slightly backwards.
- Breathe out through the mouth.
- Put mouthpiece in mouth with lips closed tightly round it.
- Actuate pump once during inspiration.
- Remove pump from mouth.
- Hold breath for 5–10 s.
- Breathe normally through the nose.
- Wait approximately 30–60 s before the next inhalation.
- Replace the cap.

For the educational intervention, W.W. explained the correct technique by referring to each illustrated step in the study leaflet and then demonstrating the correct technique. Patients were given a copy of the leaflet in their chosen language and were encouraged to refer to it at home to reinforce the information learnt during the intervention. They were asked to return in 4 weeks for the follow-up interview during which MDI use was assessed and patient acceptability of the study leaflet was evaluated. Comprehension of all pictograms in the leaflet was individually assessed.

### Data analysis

Frequency data were generated for all variables. Chi-squared tests, using the McNemar–Bowker test for matched pairs analysis, were used to test for differences between baseline and follow-up data. Pearson correlation analysis was conducted to determine if there was an association between the mean number of steps performed correctly (at baseline) with duration of MDI usage. The influence of age, education and gender on acceptability of the leaflet was assessed using Pearson chi-squared tests. The level of significance was set at *p* < 0.05.

### Ethical considerations

Ethics approval was obtained from the Rhodes University Pharmacy Ethics Committee [PHARM-2012-10] and permission to conduct the study was granted from the Eastern Cape Department of Health and Cacadu District Municipality.

## Results

### Demographic characteristics

A total of 57 patients were interviewed at baseline, with 55 returning for follow-up. The majority of patients were female (69.1%), and the age range was 18–86 years with a mean age of 58.9 ± 14.2 years (Table 1). All but one patient were black African, with isiXhosa as their first language. Just under half (47.3%) had no formal education, and a further third (34.5%) only had primary school education. The mean number of years of formal schooling was 3.0 ± 3.8 years. Most (83.6%) were unemployed.

**TABLE 1:** Demographic characteristics and self-reported metered dose inhaler use (*n* = 55).

Variables	Total	
	*n*	%
**Gender**		
Male	17	30.9
Female	38	69.1
**Age (years)**		
< 50	12	21.8
50–59	17	30.9
60–69	12	21.8
≥ 70	13	23.7
Unable to remember	1	1.8
**Ethnicity**		
Black	54	98.2
White	1	1.8
**Highest level of education**		
No formal education	26	47.3
Grades 1–7	19	34.5
Grades 8–12	10	18.2
**Home language**		
isiXhosa	54	98.2
Afrikaans	1	1.8
**Employment**		
Unemployed	46	83.6
Part time	5	9.1
Full time	4	7.3
**Duration of asthma (years)**		
< 5	12	21.8
5–20	18	32.7
21–40	16	29.1
> 40	9	16.4
**Duration of MDI usage (years)**		
< 2	11	20.0
2–5	15	27.3
> 6	29	52.7
**Prescribed MDI**		
Salbutamol	53	96.3
Beclomethasone	44	80.0
**MDI use demonstrated**		
Yes	48	87.3
**Who demonstrated MDI use (*n* = 48)**		
Doctor	34	70.8
Nurse	11	22.9
Pharmacy assistant	1	2.1
Friends or family	2	4.2

MDI, metered dose inhaler.

The mean self-reported duration of asthma was 21.8 ± 20.6 years, and mean duration of MDI usage was 7.6 ± 7.1 years. The majority (87.3%) responded that they had been previously shown how to use MDIs, with most (70.8%) having been shown by a doctor.

### Salbutamol metered dose inhaler usage and opinion of its role in symptom management

From Table 2, the majority of patients responded that salbutamol did help with managing asthma symptoms (baseline 90.6%; follow-up 94.6%). Reported number of inhalations at each MDI usage ranged from one to four, with the most common dose being two inhalations. There was no significant change in frequency of use of the MDI between baseline and follow-up, with the majority in both phases responding that they used salbutamol only when required (*p* = 0.070). The number of patients who had been prescribed salbutamol increased from 53 at baseline to 55 at follow-up as a result of recommendations from W.W. after the baseline interviews.

**TABLE 2:** Self-reported usage and opinion of salbutamol metered dose inhaler.

Question	Baseline (*n* = 53)		Follow-up (*n* = 55)		*p*
	*n*	%	*n*	%	
**Does salbutamol help your asthma?**					0.625
Yes	48	90.6	52	94.6	
No	0	0.0	1	1.8	
Unsure	5	9.4	2	3.6	
**How many puffs do you use each time?**					0.117
One	7	13.2	4	7.3	
Two	34	64.2	36	65.5	
Three	9	17.0	7	12.7	
Four	3	5.6	8	14.5	
**Do you ever take more puffs than you stated?**					0.078
Yes	35	66.0	26	47.3	
No	18	34.0	27	49.1	
No response	0	0.0	2	3.6	
**How often do you use it each day?**					0.070
When required	46	86.8	53	96.4	
Twice a day	4	7.5	1	1.8	
Three times a day	3	5.7	1	1.8	

### Demonstration of metered dose inhaler technique at baseline and follow-up

**FIGURE 2:**
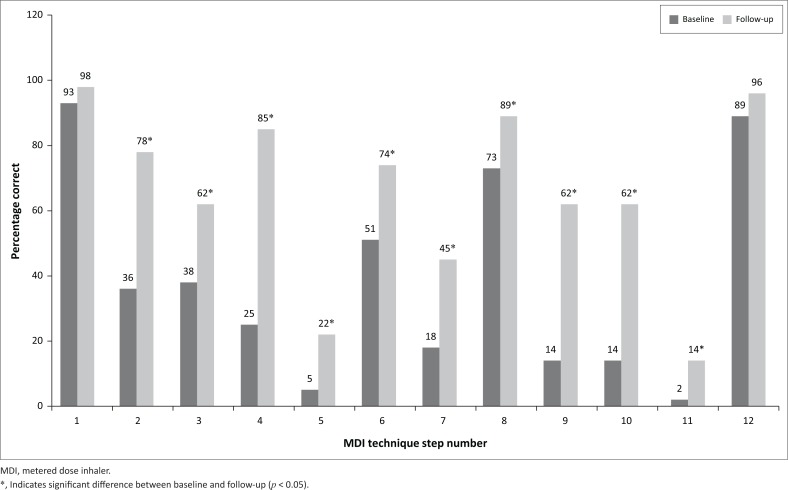
Comparison of correct usage of metered dose inhaler for each step.

To evaluate the effect of the educational intervention on correct MDI use, Figure 2 shows a comparison of the baseline and follow-up patient demonstrations. A significant improvement of the correct technique was evident in 10 of the 12 steps. The only steps showing no significant improvement were the first and last steps (remove or replace the cap) as the majority of patients demonstrated these correctly in both phases.

Prior to the intervention, no patients demonstrated all 12 steps correctly; at follow-up after the intervention, only one patient was able to correctly demonstrate all 12 steps. The mean number of correct steps increased significantly from 4.6 ± 2.2 at baseline to 7.9 ± 2.7 at follow-up. A weak, positive correlation between the number of years using MDIs and the mean number of correct steps at baseline was established (*r* = 0.28; *n* = 54; *p* = 0.039).

Table 3 shows the most common errors in MDI usage. Failing to wait for 30–60 seconds between inhalations and failing to exhale before placing the MDI in the mouth were recorded as the two steps with the highest frequency at both baseline and follow-up.

**TABLE 3:** Metered dose inhaler technique steps incurring the highest number of incorrect responses (*n* = 55).

MDI step		Patients	
		*n*	%
**Baseline**			
11.	Wait 30–60 seconds before next inhalation	54	98.2
5.	Breathe out through mouth before putting mouthpiece in mouth	52	94.6
9.	Hold breath for 5–10 seconds after inhalation	47	85.5
10.	Breathe normally through the nose	47	85.5
7.	Actuate pump once during inspiration	45	81.8
4.	Tilt head slightly backwards	41	74.6
**Follow-up**			
11.	Wait 30–60 seconds before next inhalation	47	85.5
5.	Breathe out through mouth before putting mouthpiece in mouth	43	78.2
7.	Actuate pump once during inspiration	30	54.6
3.	Shake pump	21	38.2
9.	Hold breath for 5–10 seconds after inhalation	21	38.2
10.	Breathe normally through the nose	21	38.2

MDI, metered dose inhaler.

### Comprehension and acceptability of the manufacturer and study leaflets

Table 4 shows that although almost all patients (52/55) reported having had a look at the manufacturer leaflet accompanying their MDI, only six said they had read it and, of these, only one claimed to have understood the written information. Over half (58.2%) stated lack of reading ability as the reason for not reading the package insert, whereas eight reported that this was because of the insert not being in their home language. Other problems included small font size, poor eyesight and never having been encouraged to read it. Although most patients said that they had seen the pictograms in the leaflet, just over half (56.4%) claimed that they were able to interpret them.

**TABLE 4:** Comprehension and acceptability of manufacturer and study leaflets (*n* = 55).

Question	Patients	
	*n*	%
**Manufacturer leaflet**		
Looked at leaflet	52	94.6
Read the text	6	10.9
Understood the text	1	1.8
Understood the pictures	31	56.4
Reasons for not reading manufacturer leaflet		
-Unable to read	32	58.2
-Text not in home language	8	14.6
-Others	9	16.3
**Study leaflet**		
Looked at leaflet	55	100.0
Read the text	26	47.3
Understood the text	26	47.3
Font size large enough	25	45.5
Understood the pictures†	48	87.3
Liked the pictures in the leaflet	54	98.2
Leaflet assisted in using MDI correctly	53	96.4
Non-patients (family, friends) read leaflet	45	81.8
Other ways in which the leaflet helped	54	98.2

† , Comprehension of pictures was formally tested. All other results are self-reported.

MDI, metered dose inhaler.

All patients had been shown the study leaflet, with just under half reporting that they could understand it (47.3%), and noting that the font size was large enough to read easily (45.5%). All but one patient approved the pictograms in the leaflet and felt that the leaflet had helped them in the correct use of their MDI. One patient commented: ‘I need to learn more especially about how to use my pump.’ Interestingly, a large number of patients reported that a family member had also wanted to read the study leaflet.

Age and education of this study population were significantly related with patients in the three older categories having significantly less education than those less than 50 years old (*p* < 0.01). Of the 26 patients who responded that it was easy to comprehend the leaflet, higher education levels, as anticipated, significantly increased readability and comprehension of the study leaflet (*p* = 0.001). Age was also a factor, with patients more than 50 years old reporting significantly greater difficulty reading and understanding the study leaflet compared to the younger age group (*p* = 0.005). These older patients also considered the font size as being too small, and had greater difficulty interpreting the pictograms. The majority of patients who reported that family members had read the leaflet were in this older age group of >50 years, with no education.

## Discussion

To our knowledge, this is the first reported study from Africa in which new pictograms for an illustrated MDI use leaflet have been designed specifically for the local population taking into account physical and cultural characteristics, as well as limited literacy skills. The success of the educational intervention is evident in the statistically significant increase in the number of correct steps from 4.6 at baseline to 7.9 at follow-up, and the statistically significant improvement in MDI technique at follow-up for 10 of the 12 steps, with the remaining two being mostly correct at baseline.

Study patients reported that inhaler technique was not regularly demonstrated by HCPs at the clinic. Baseline technique was poor, with only four of the steps achieving more than 50% correct at baseline despite most patients reporting that they had been shown how to use their MDI by an HCP. As 80% of study patients had been using their inhalers for more than 2 years, this is a disconcerting finding as incorrect MDI use results in inadequate treatment outcomes, and poorer control of asthma symptoms. Although correct technique increased significantly after the intervention, the likelihood of this improvement being sustained is uncertain. Regular reinforcement of the complex MDI technique is of utmost importance to maintain changed patient behaviour,^35^ with practical demonstration known to be the best method to educate patients and to reduce usage errors associated with MDI use.^23,36^

The impact of regular reinforcement of MDI technique is enhanced when a standardised checklist is included.^37^ Unfortunately, a checklist was not standard practice at the study clinic, nor were we able to establish whether a placebo MDI was available for demonstration purposes. This calls into question the likelihood of patients receiving consistently correct MDI demonstrations of each step. It appeared that the patient’s own MDI was used for demonstration, raising the possibility that, during the demonstration, the HCP may have avoided inhaling the active ingredient by not placing the mouthpiece in the mouth, and failing to demonstrate actuation with the MDI in the proper position. Even if these steps had been verbally explained, the absence of a physical demonstration of the correct technique is unlikely to successfully educate patients. Findings from a recent systematic review by Usmani et al. clearly illustrated that inhaler technique can be affected by the level of instruction from HCPs.^4^

Giraud and Roche^18^ found that although 71% of MDI users had co-ordination errors, only 15% rated themselves as poor or very poor users, thereby displaying false confidence in their user ability. This suggests that merely questioning about correct inhaler use is unlikely to be adequate, with our findings demonstrating that formal assessment of MDI technique is essential to ensure that incorrect technique is corrected in order to ensure optimal treatment outcomes.

Leaflets containing pictograms have been shown to result in improved patient recall, understanding of information and adherence in low-literacy populations.^38^ Almost all patients in our study (96.4%) reported that the leaflet had helped with their MDI technique, and many commented that the demonstration of MDI technique by W.W. had also been beneficial. Patients who were able to read commented positively on the simplicity of the leaflet. One patient whose home language was Afrikaans said that he had no problems reading the English leaflet as the language was ‘easy, plain English’. Even though almost half the population had no formal schooling, which could be linked with poor visual literacy skills, 54 of the 55 study patients expressed their approval of the pictograms designed for the leaflet, as the pictograms contained visual elements more familiar to them than other leaflets.

An Italian study identified that reading of the manufacturer’s package insert was significantly associated with both higher education and correct MDI technique.^11^ The current study, with its patient population having a low average level of education, found that only 1 of 55 patients reported understanding the text in the manufacturer leaflet, although six patients stated that they had read the leaflet. It was interesting to note that the average education level of the six who had read the manufacturer leaflet was grade 9, markedly higher than the study average of grade 3. As patients often use the manufacturer information to recall and reinforce information that is verbally communicated by HCPs, the inability to read the information could be one reason for their poor MDI technique.

The design of the study leaflet was grounded in the literature, but also involved wide consultation with a variety of health professionals and, very importantly, was also a user-centred process. This process ensured that the textual content was validated by health professionals, whereas consultation with end users from the target population ensured cultural validity, an acceptable readability level and visual content that was relevant and familiar to the user population. All this contributed to the acceptability of the leaflets, and, in many cases, to their wider distribution by patients to members of the community.

Evidence shows that individualised asthma self-management plans improve medication adherence and markers of asthma control.^39^ However, the under-resourced nature of the health care system in South Africa^40,41^ suggests that regular reviewing and implementation of personalised asthma management plans is unlikely to occur for all patients. The study leaflet has the potential to serve as a tool to reduce the time needed to write individual management plans, as specific problem areas could be highlighted, which would serve as a reminder for the patient and other family members or supporters. The important role of nurses who have a good knowledge of asthma in evaluating asthma patients and in providing patient education has been established.^42,43^ Primary health care clinics in South Africa are staffed mainly by nurses and are assisted by community health workers, both of whom could use the study leaflet as a training tool to educate patients about MDI usage. Many study patients reported that a family member had also read the leaflet, attracted by its visual content and simplicity. In traditional rural communities from which this study population is drawn, management of health problems and medicine use is often a communal issue, with family members playing an important role in supporting their family members. This is particularly important where the children who are educated are able to read health information and assist the older generations in their medicine-taking practice.

### Strengths and limitations of the study

Limitations of the study included the use of only one clinic site for this main study, with all but one patient being drawn from the same ethnic and language group, limiting generalisability to the broader South African population or to other countries. Sample size was small and was limited by the total number of asthma patients in the surrounding deeply rural area.

The same researcher (W.W.) collected all the data, contributing to the strength of the study. A further strength is the multi-phase iterative design process adopted for pictogram and leaflet design which, combined with wide consultation, resulted in constant changes and improved versions, and enhanced the acceptability of the final leaflet.

### Implications for practice

The study leaflet could be considered as an online repository for use by HCPs, community health workers and patients, with the following guidelines proposed for optimal use:

- Health care professionals using illustrated leaflets should offer a clear, verbal explanation in the patient’s home language to clarify and reinforce the written information, and should physically demonstrate MDI technique.
- Regular demonstration of MDI technique with a placebo MDI, as well as using a standardised checklist to identify any errors requiring correction reflects best practice. The checklist should be placed in the patient folder to allow for reference at the following visit.
- Patients must always be given an opportunity to ask HCPs questions to clarify any uncertainties.

## Conclusion

An educational intervention consisting of the newly designed illustrated study leaflet in combination with a demonstration of MDI technique resulted in a significant increase in correct MDI technique in low-literacy asthma patients. The patient-centred approach adopted for the design of the leaflet components, along with consultation with a broad range of HCPs in the iterative development process contributed to cultural relevance and content validity of the leaflet. Acceptability of the leaflet and all its components was high. However, the study results also indicate the need for ongoing patient education to further improve and to regularly reinforce MDI technique.
